# Association between novel obesity- and lipid-related indices and diabetes across different FBG among elderly: a prospective cohort study

**DOI:** 10.3389/fendo.2026.1798687

**Published:** 2026-03-26

**Authors:** Xiang-yang He, Dong-hui Ma, Zheng Liu, Yan-Fang Guo, Ren-cheng Zhao, Pan-pan Sun, Xing-lin Zhong

**Affiliations:** 1 Department of Health Management, Shenzhen Baoan District Chronic Diseases Prevent and Cure Hospital, Shenzhen, Guangdong, China; 2 Department of Endocrinology, The 987^th^ Hospital of the PLA Joint Logistics Support Force, Baoji, Shaanxi, China; 3 Medical Department, Shenzhen University of Advanced Technology General Hospital, Shenzhen, Guangdong, China

**Keywords:** type 2 diabetes (T2D), prospective cohort study, elderly, visceral adiposity index (VAI), lipid accumulation product (LAP), triglyceride glucose (TyG), triglyceride high-density cholesterol-glucose body (TyHGB)

## Abstract

**Background:**

Although VAI, LAP, TyG and TyHGB are considered alternative indicators of abdominal fat deposition, their longitudinal association and strength of these indices with T2D remain unclear. The study aimed to evaluate the association between the indices and onset T2D in the elderly population across different FBG statuses and compare their predictive performance in risk assessment.

**Methods:**

Data were from the BaHLS, a cohort study of community-dwelling elderly individuals in Shenzhen, China. The study examined the associations between six novel obesity- and lipid-related indices (including BMI, WC, VAI, LAP, TyG and TyHGB) and the onset of T2D across different FBG statuses, including normal and elevated FBG. A multivariate *Cox* proportional hazards model and a *GAM* were employed to assess the longitudinal associations between each index and T2D. *ROC* curves analysis and *AUC* were utilized to evaluate the predictive performance of indices and determine their optimal cutoff values.

**Results:**

A total of 18,251 participants were enrolled in the study, with 1,350 (7.40%) participants were diagnosed with T2D by end of 2022. In the overall elderly cohort, the adjusted *HRs* of T2D per one-standard-deviation increase were: BMI 1.17 (95% *CI*: 1.11–1.23), WC 1.23 (95% *CI*: 1.16–1.30), VAI 1.30 (95% *CI*: 1.24–1.37), LAP 1.21 (1.17–1.26), TyG 1.76 (95% *CI*: 1.67–1.86), and TyHGB 1.37 (95% *CI*: 1.33–1.42). Except for BMI and WC, the remaining indices were independent risk factors for the onset of T2D in both normal and elevated FBG individuals. These indices showed significant associations with T2D across different subgroups. Regarding predictive performance, all six indices demonstrated predictive ability for the onset of T2D across all participants (*AUC* > 0.5, *P<* 0.001). Specifically, TyG (*AUC* = 0.706, 95% *CI*: 0.691–0.721) and TyHGB (*AUC* = 0.737, 95% *CI*: 0.724–0.750) exhibited better predictive performance than the other indices. *RCS* analysis revealed that BMI and WC exhibited linear associations with the risk of T2D across all participants, whereas VAI, LAP, TyG, and TyHGB demonstrated nonlinear relationships.

**Conclusion:**

The study demonstrated that 6 obesity- and lipid- indices are positively associated with the incidence of onset T2D, and TyG and TyHGB demonstrated superior predictive performance for the onset of T2D in the elderly. Suggesting that TyG and TyHGB should be served as a valuable indicator for monitoring and preventing T2D.

## Introduction

T2D has emerged as a significant public health concern, with a worldwide adult prevalence of 10.5% and a particularly alarming growth rate among older adults (1, 2). It is especially relevant considering global aging trends, individuals aged 65 and older constituted 9.0% globally, while in China, this demographic represented 14.2% in 2020. Projections indicate that the elderly population in China will reach 395 million by 2025 (3, 4). Individuals with T2D face a two-fold increased risk of CVD compared to those without (5). Furthermore, T2D can lead to debilitating complications such as blindness and kidney failure, placing a substantial economic burden on healthcare systems (6, 7). Consequently, the development of efficient and accessible screening tools to identify individuals at an early stage is crucial for enabling early intervention and management.

Epidemiological studies have indicated that obesity is a significant risk factor for T2D, playing a crucial role in the onset of insulin resistance and progression of the disease (8, 9). It is frequently used in clinical practice to identify individuals at high risk for T2D. Currently, It is the most widely used measure of obesity and has been validated by a meta-analysis of Mendelian randomization studies, to be associated with a higher risk of T2D (10). Nevertheless, the primary factor influencing cardiovascular metabolic disease risk is the distribution of fat tissue rather than the overall level of obesity (11). BMI cannot differentiate between lean body mass and total fat mass, nor can it capture the patterns of fat distribution (12, 13). Consequently, several studies have adopted novel obesity- and lipid-related indices—such as WC, VAI, LAP, TyG, and TyHGB—to assess visceral fat distribution. These measures are considered superior indices of cardiovascular metabolic risk (14–17).

Currently, studies investigating the association between obesity- and lipid-related indices and T2D are limited, and their conclusions remain inconsistent. For example, some research suggests that WC is a better predictor of T2D risk than BMI in Western populations (18, 19), although evidence remains controversial in Asian populations (20, 21). Additionally, studies report inconsistent predictive efficacy of LAP, VAI, and TyG for T2D (22–24). While some studies have identified a linear association between the TyG index and T2D (24, 25), no longitudinal studies have yet examined the relationship between the TyHGB and T2D. In summary, no definitive conclusion currently exists regarding which indicator offers the greatest predictive advantage for T2D. Most previous studies have focused on Western populations or younger cohorts, which may contribute to inconsistent results due to differences in age, race, and culture. Furthermore, the longitudinal association between these indices and T2D may vary across different glycemic statuses. Therefore, further research is needed to clarify the relationship between obesity- and lipid-related indices and T2D in elderly populations with varying glycemic statuses, to better inform the prevention and management of T2D in this group.

## Method

### Data sources and study population

The study analyzed data from the BaHLS in Shenzhen, China. which focused on the elderly population in Shenzhen, China (26). BaHLS is a long-term study involving individuals aged 60 and above, having enrolled 22,507 participants between January and December 2020 (Wave 0). Participants undergo an annual structured interview and health assessment. Three follow-up surveys have been competed: Wave 1 in 2021 and Wave 2 in 2022.

**Figure 1:**
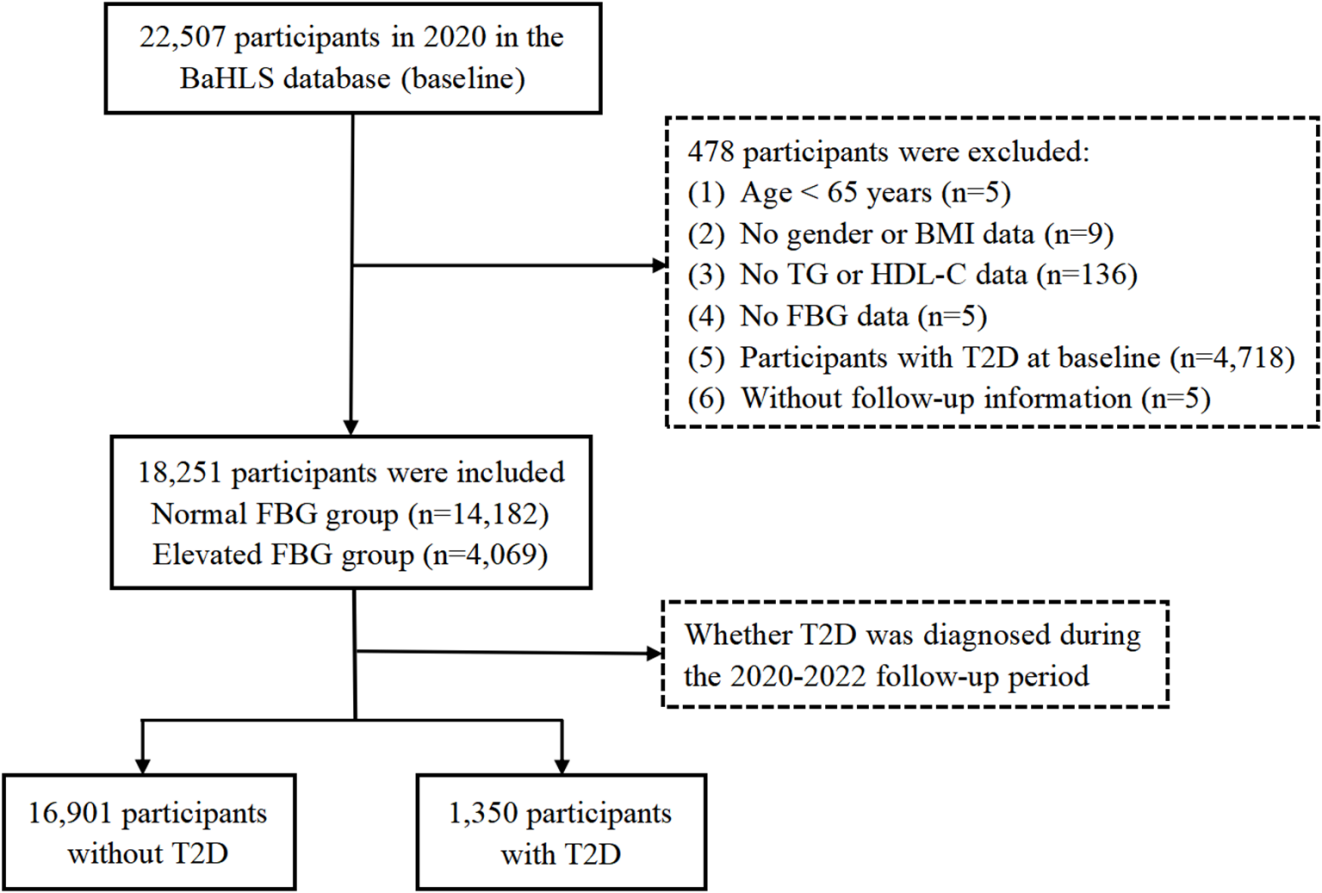
Flowchart of participants selection from BaHLS 2020–2022.

Among the 22,507 baseline participants in 2020, 4,718 were diagnosed with T2D. Additional exclusions were made for those under 60 years old (n = 5), those with incomplete data (n = 150), and those without follow-up information (n = 5). Ultimately, 18,251 participants with complete data were included in the analysis, comprising of 14,182 individuals with normal FBG levels and 4,069 with elevated FBG levels. Elevated FBG was defined as FBG levels between 6.1 mmol/L and less than 7.0 mmol/L, while normal FBG was defined as FBG levels below 6.1 mmol/L. **Figure 1** provides a flowchart detailing the criteria for participant inclusion and exclusion.

### Data collection

All data were gathered by trained healthcare professionals. The main sources of data included standardized questionnaires, physical assessments, and collection of biological samples. The questionnaires mainly addressed sociodemographic information (such as age, sex, and education), lifestyle habits (including smoking, alcohol use, and physical activity), medical history, and family medical background. Physical assessments adhered to WHO standard procedures (27), measuring participants’ height, weight, waist circumference, and blood pressure. Height and weight were taken with participants barefoot and in light clothing; height was recorded to the nearest 0.1 cm and weight to the nearest 0.1 kg. Waist circumference was measured with a 1.5-meter tape at the midpoint between the lower rib margin and the iliac crest along the mid-axillary line. Blood pressure was measured twice using a calibrated mercury sphygmomanometer after participants had rested seated for 10 minutes, and the average of the two readings was used for analysis (28). For biological sampling, participants fasted for at least 8 hours before venous blood was drawn by a nurse. Blood samples were analyzed within 4 hours of collection. TC, TG, LDL-C, and HDL-C were measured using an automated biochemical analyzer. TC and TG were assessed using enzymatic methods with commercially available reagents, while HDL-C and LDL-C were measured by a timed-endpoint colorimetric technique. FBG was determined using the glucose oxidase method (29).

### Measurements

Obesity- and lipid-related indices were calculated based on participants’ weight, height, WC, TG, HDL, and FBG, including BMI (30), VAI (31), LAP (23), TyG, and TyHGB (32). The formulas for these indices were:


$$\text{BMI }=\text{ weight }\left(\text{kg}\right)/{\text{height}}^{2}\left(\text{m}\right)$$

$$\text{VAI }\left(\text{male}\right)=\left[\frac{\text{WC }\left(\text{cm}\right)}{39.68+1.88\times \text{BMI}(\text{kg}/{\text{m}}^{2})}\right]\times \left[\frac{\text{TG}(\text{mmol}/\text{L})}{1.03}\right]\times \left[\frac{1.31}{\text{HDL}(\text{mmol}/\text{L})}\right]$$

$$\text{VAI }\left(\text{female}\right)=\left[\frac{\text{WC }\left(\text{cm}\right)}{36.58+1.89\times \text{BMI}(\text{kg}/{\text{m}}^{2})}\right]\times \left[\frac{\text{TG}(\text{mmol}/\text{L})}{0.81}\right]\times \left[\frac{1.52}{\text{HDL}(\text{mmol}/\text{L})}\right]$$

LAP (male)=[WC(cm)−65]×TG(mmol/L)

LAP (female)=[WC(cm)−58]×TG(mmol/L)

TyG index=Ln[TG(mg/dL)×FBG(mg/dL)/2]

$$\text{TyHGB}=\text{TG}\left(\text{mg}/\text{dL}\right)/\text{HDL}-\text{C}\left(\text{mg}/\text{dL}\right)+0.7\times \text{FBG}(\text{mmol}/\text{L})+0.1\times \text{BMI}(\text{kg}/{\text{m}}^{2})$$

### Follow-up and outcomes assessment

Each follow-up assessment for participants was conducted by healthcare professionals affiliated with the same medical institution, encompassing both interviews and physical examinations as previously described. The primary outcome variable was the incidence of T2D diagnosed during the follow-up period. In accordance with the criteria established by the American Diabetes Association (33), T2D diagnosis was confirmed if one or more of the following conditions were satisfied: (1) FBG level exceeding 7.0 mmol/L; (2) random blood glucose level greater than 11.1 mmol/L; (3) HbA1c level above 6.5%; (4) self-reported physician diagnosis of diabetes, with verification of the diagnosis date; (5) current use of hypoglycemic treatments, including both traditional Chinese medicine and contemporary Western pharmacotherapy; or (6) ongoing treatment for diabetes or hyperglycemia with other therapeutic modalities.

### Potential confounding variables

This study controlled for baseline sociodemographic characteristics, lifestyle behaviors, physical measurements, and selected biological indicators that may potentially affect diabetes risk, incorporating these factors as covariates. The sociodemographic variables encompassed gender, age, marital status (categorized married or other), educational attainment (classified Primary school and below, middle school, or high school and above), family history of diabetes, and history of hypertension. History of hypertension was self-reported, defined as either a prior physician diagnosis or current use of antihypertensive medication. Lifestyle behaviors included smoking status (never smoked, current smoker, or former smoker) and alcohol consumption (current drinker or non-drinker). Physical measurements consisted of SBP and DBP, and among other parameters. The biological indicators primarily included hemoglobin, TC, and LDL.

### Statistical analysis

Participants were stratified into two groups according to baseline FBG levels: the normal FBG group (FBG< 6.1 mmol/L) and the elevated FBG group (6.1 mmol/L ≤ FBG< 7.0 mmol/L). Baseline measurements of BMI, WC, VAI, LAP, TyG, and TyHGB measurements were subsequently categorized into quartiles. Baseline characteristics were compared according to the diagnosis of T2D during follow-up period. Continuous variables with normal distribution were presented as means± standard deviation, and between-group comparisons were conducted using *t-tests*. For continuous variables exhibiting non-normal distributions, *IQRs* were reported, with group comparisons performed via the Kruskal-Wallis test. Categorical variables were expressed as frequencies and percentages, and intergroup differences were assessed using chi-square tests. Covariates incorporated into the analyses included traditional and potential risk factors that may influence the development of T2D.

For the analysis of longitudinal follow-up data, *Cox* proportional hazards models were utilized to estimate *HRs* and 95% *CI* to examine the association between obesity- and lipid-related indices and the occurrence of T2D events. Three models were constructed: Model 1 included unadjusted; Model 2 adjusted for demographic and lifestyle factors including age, sex, education level, marital status, smoking status, alcohol consumption, and family history of diabetes; Model 3 further adjusted for clinical variables such as hemoglobin levels, hypertension status, SBP, DBP, TC, and LDL-C into Model 2. The Kaplan-Meier method was employed to compare cumulative incidence rates across different subpopulations. The predictive performance of each index for incident T2D was evaluated using *ROC* curve analysis, with optimal cutoff values determined by the Youden index. Additionally, *GAM* incorporating *RCS* were applied to assess the dose-response relationships between obesity- and lipid-related indices and the risk of onset T2D. Subgroup analyses were performed to investigate these associations within strata defined by age groups (65–75 years and ≥75 years), sex, and hypertension status (presence or absence). Finally, sensitivity analyses were conducted by excluding participants who died during the follow-up period to verify the robustness of the findings.

All the statistical analyses in the study were conducted using IBM SPSS (Version 26.0) and R version 4.4.1 (R Foundation for Statistical Computing). *P* < 0.05 was considered statistically significant in the study.

## Results

### Baseline characteristics of 18295 participants

The study cohort comprised 18,251 individuals without T2D at baseline, including 10,212 females (55.95%) and 8,039 males (44.05%). The mean age of participants was 66.04 ± 4.86 years, ranging from 60 to 99 years. Among the subjects, 1,888 (10.34%) were current smokers, 1,879 (10.30%) were current alcohol consumers, and 52.11% had a diagnosis of hypertension. Based on baseline FBG levels, participants were stratified into two groups: a normal FBG group (n = 14,182) and a elevated FBG group (n = 4,069). Participants with elevated FBG exhibited significantly higher mean age, BMI, WC, SBP, DBP, TC, TG, VAI, LAP, TyG, and TyHGB levels compared to those with normal glucose. Additionally, the elevated FBG group had higher proportions of female, lower education levels, current smokers, current alcohol consumers, with hypertension, and with diabetic family history (*p* < 0.05). No significant differences were identified in other characteristics (*p* > 0.05). Detailed participant characteristics are presented in **Table 1**.

**Table 1:** Basic characteristics of participants at baseline in 2020.

Characteristics	Total (n=18,251)	Normal FBG (n=14,182)	Elevated FBG (n=4,069)	*P value*
Age ^a^, years	66.04 ± 4.86	65.99 ± 4.83	66.51 ± 4.98	0.015
Waistline ^a^, cm	86.38 ± 8.73	85.81 ± 8.68	88.40 ± 8.62	<0.001
Female ^c^ , *n*(%)	10,212 (55.95)	7,839 (55.27)	2,373 (58.32)	0.001
Married ^c^ , *n*(%)	1,7720 (97.09)	13,779 (97.16)	3,941 (96.85)	0.309
BMI ^ac^, kg/m^2^	23.99 ± 3.14	23.78 ± 3.10	24.74 ± 3.17	<0.001
<25	11759 (64.43)	9509 (67.05)	2250 (55.30)	<0.001
≥25	6492 (35.57)	4673 (32.95)	1819 (44.70)	
Education level ^c^ , *n*(%)				
Primary school and below	9,996 (54.77)	7,645 (53.91)	2,351 (57.78)	<0.001
Middle school	5,567 (30.50)	4,381 (30.89)	1,186 (29.15)	
High School or above	2,688 (14.73)	2,156 (15.20)	532 (13.07)	
Smoking ^c^ , *n(%)*				
never smoker	14,555 (79.75)	11,293 (79.63)	3,262 (80.17)	0.001
current smoker	1,888 (10.34)	1,524 (10.75)	364 (8.95)	
former smoker	1,808 (9.91)	1,365 (9.62)	443 (10.89)	
Alcohol consumption ^c^ , *n(%)*				
non-drinker	16,372 (89.70)	12,755 (89.94)	3,617 (88.89)	0.053
current drinker	1,879 (10.30)	1,427 (10.06)	452 (11.11)	
Family history of diabetes ^c^ , *n(%)*	577 (3.16)	400 (2.82)	177 (4.35)	<0.001
With hypertension ^c^ , *n(%)*	9,511 (52.11)	7,085 (49.96)	2,426 (59.62)	<0.001
Laboratory and Clinical Characteristics				
SBP ^a^, mmHg	133.97 ± 16.99	133.30 ± 17.02	136.50 ± 16.65	<0.001
DBP ^a^, mmHg	78.50 ± 9.92	78.27 ± 9.95	79.29 ± 9.78	<0.001
Hb ^a^, g/dL	13.66 ± 1.54	13.61 ± 1.52	13.84 ± 1.60	<0.001
FBG ^a^, mmol/L	5.44 ± 0.77	5.12 ± 0.54	6.53 ± 0.28	<0.001
TC ^a^, mmol/L	5.10 ± 1.10	5.07 ± 1.08	5.17 ± 1.13	<0.001
TG ^a^, mmol/L	1.57 ± 0.95	1.51 ± 0.90	1.81 ± 1.10	<0.001
HDL-C ^a^, mmol/L	1.36 ± 0.38	1.37 ± 0.38	1.32 ± 0.37	0.001
LDL-C ^a^, mmol/L	2.94 ± 0.93	2.93 ± 0.92	2.94 ± 0.95	0.708
Obesity- and lipid-related indices				
VAI ^b^	2.01 (1.32, 3.07)	1.92 (1.25, 2.90)	2.37 (1.59, 3.64)	<0.001
LAP ^b^	33.75 (21.00, 52.52)	31.74 (19.60, 49.28)	41.92 (27.12, 64.09)	<0.001
TyG ^a^	8.68 ± 0.54	8.59 ± 0.51	9.01 ± 0.52	<0.001
TyHGB ^a^	9.15 ± 2.48	8.76 ± 2.28	10.50 ± 2.65	<0.001

a *t* test; b Kruskal–Wallis test; c chi-square test.

BMI, body math index; SBP, systolic blood pressure; DBP, diastolic blood pressure; Hb, hemoglobin; FBG, fasting blood glucose; TC, total cholesterol; TG, triglyceride; HDL-C, high-density lipoprotein cholesterol; LDL-C, low-density lipoprotein cholesterol; VAI, visceral adiposity index; LAP, lipid accumulation product; TyG, triglyceride glucose index; TyHGB, triglyceride high-density cholesterol-glucose body index.

### Characteristics associated with the development of T2D

During the follow-up period, 1,350 participants (7.40%) were diagnosed with T2D. Compared to those without T2D, individuals who developed T2D demonstrated significantly higher baseline levels of Hb, TG, BMI, WC, VAI, LAP, TyG, and TyHGB, Additionally, the prevalence of hypertension was greater among the T2D group (70.89% versus 50.61%), as was the incidence of a family history of diabetes (9.89% versus 2.86%). Conversely, TC and HDL-C levels were lower in the T2D cohort, alongside a reduced proportion of married individuals (95.85% versus 97.19%). All observed differences reached statistical significance (*p* < 0.05). Detailed data are presented in **Table 2**.

**Table 2:** Characteristics of participants at baseline that developed T2D and those that did not.

Characteristics	Total (n=18,251)	Without T2D (n=16,901)	With T2D (n=1,350)	*P value*
Age ^a^, years	66.04 ± 4.86	66.02 ± 4.84	66.26 ± 5.17	0.082
Waistline ^a^, cm	86.38 ± 8.73	86.21 ± 8.70	88.61 ± 8.89	<0.001
Female ^c^ , *n*(%)	10,212 (55.95)	9430 (55.80)	782 (57.93)	0.129
Married ^c^ , *n*(%)	1,7720 (97.09)	16426 (97.19)	1294 (95.85)	0.005
BMI ^a^ , kg/m^2^	23.99 ± 3.14	23.94 ± 3.13	24.69 ± 3.26	<0.001
Education level ^c^ , *n*(%)				
Primary school and below	9,996 (54.77)	9,220 (54.55)	776 (57.48)	0.082
Middle school	5,567 (30.50)	5,189 (30.70)	378 (28.00)	
High School or above	2,688 (14.73)	2,492 (14.74)	196 (14.52)	
Smoking ^c^ , *n(%)*				
never smoker	14,555 (79.75)	13,472 (79.71)	1,083 (80.22)	0.518
current smoker	1,888 (10.34)	1,760 (10.41)	128 (9.48)	
former smoker	1,808 (9.91)	1,669 (9.88)	139 (10.30)	
Alcohol consumption ^c^ , *n(%)*				
non-drinker	16,372 (89.70)	15,146 (89.62)	1,226 (90.81)	0.163
current drinker	1,879 (10.30)	1,755 (10.38)	124 (9.19)	
Family history of diabetes ^c^ , *n(%)*	577 (3.16)	484 (2.86)	93 (9.89)	<0.001
With hypertension ^c^ , *n(%)*	9,511 (52.11)	8,554 (50.61)	957 (70.89)	<0.001
Laboratory and Clinical Characteristics				
SBP ^a^, mmHg	133.97 ± 16.99	133.90 ± 17.06	134.80 ± 16.05	0.060
DBP ^a^, mmHg	78.50 ± 9.92	78.47 ± 9.95	78.84 ± 9.51	0.181
Hb ^a^, g/dL	13.66 ± 1.54	13.66 ± 1.53	13.74 ± 1.63	0.047
FBG ^a^, mmol/L	5.44 ± 0.77	5.37 ± 0.72	6.35 ± 0.69	<0.001
TC ^a^, mmol/L	5.10 ± 1.10	5.10 ± 1.09	5.00 ± 1.16	0.001
TG ^a^, mmol/L	1.57 ± 0.95	1.55 ± 0.94	1.82 ± 1.10	<0.001
HDL-C ^a^, mmol/L	1.36 ± 0.38	1.36 ± 0.38	1.29 ± 0.35	<0.001
LDL-C ^a^, mmol/L	2.94 ± 0.93	2.94 ± 0.63	2.90 ± 0.97	0.099
Obesity- and lipid-related indices				
VAI ^b^	2.01 (1.32, 3.07)	1.97 (1.30, 3.06)	2.40 (1.67, 3.70)	<0.001
LAP ^b^	33.75 (21.00, 52.52)	33.18 (20.58, 51.50)	42.05 (27.25, 64.17)	<0.001
TyG ^a^	8.68 ± 0.54	8.66 ± 0.53	8.98 ± 0.55	<0.001
TyHGB ^a^	9.15 ± 2.48	9.04 ± 2.41	10.47 ± 2.88	<0.001

a *t* test; b Kruskal–Wallis test; c chi-square test.

BMI, body math index; SBP, systolic blood pressure; DBP, diastolic blood pressure; Hb, hemoglobin; FBG, fasting blood glucose; TC, total cholesterol; TG, triglyceride; HDL-C, high-density lipoprotein cholesterol; LDL-C, low-density lipoprotein cholesterol; VAI, visceral adiposity index; LAP, lipid accumulation product; TyG, triglyceride glucose index; TyHGB, triglyceride high-density cholesterol-glucose body index.

### Risk of obesity- and lipid-related indices and oneset of T2D

**Figure 2:**
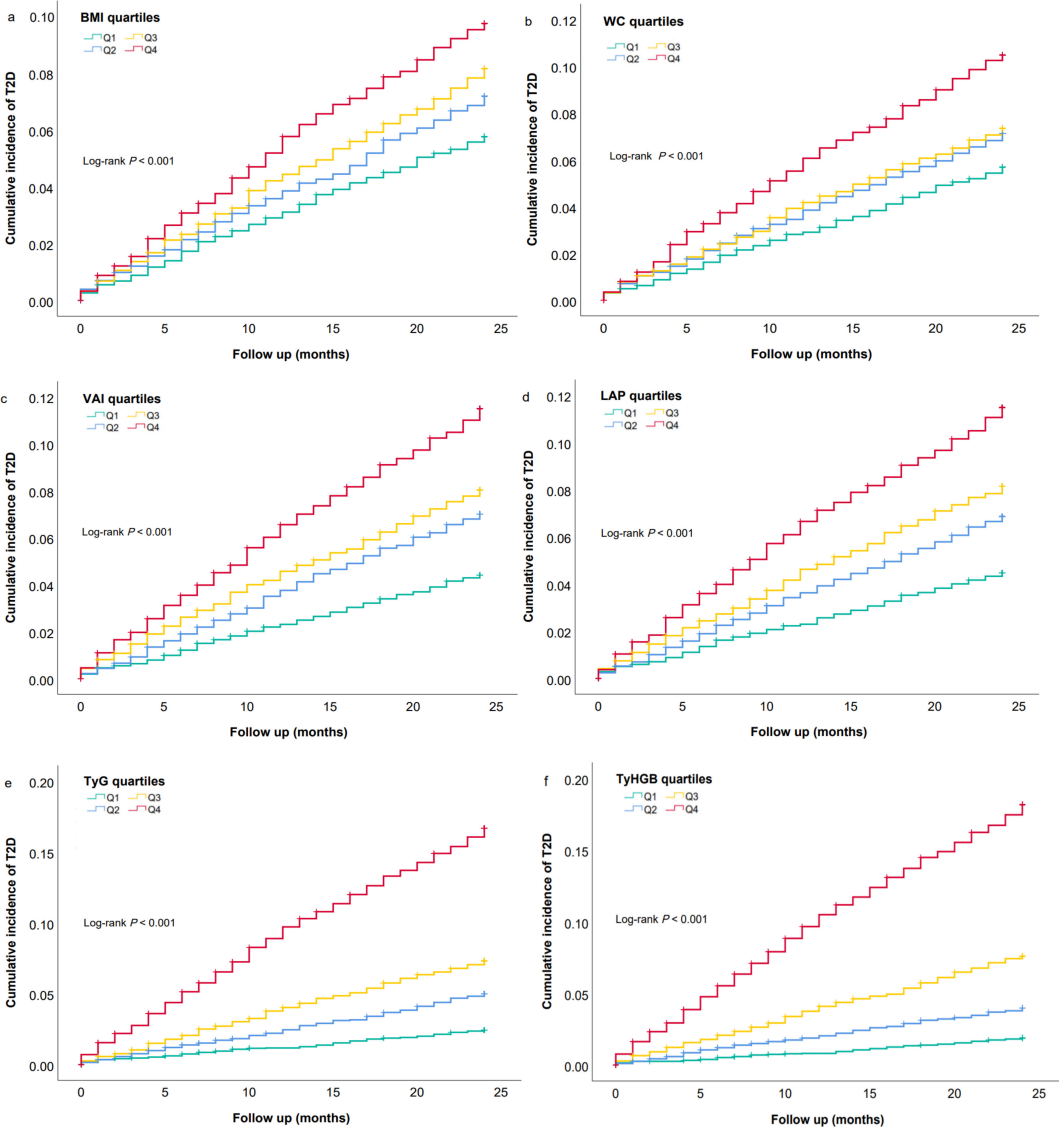
Kaplan - Meier incidence rate of T2D according to quartiles of novel obesity- and lipid-related indices among all participants. **(a)** BMI, **(b)** WC, **(c)** VAI, **(d)** LAP, **(e)** TyG, **(f)** TyHGB.

**Figure 2** displayed Kaplan-Meier survival curves depicting the risk of T2D stratified by quartiles of obesity- and lipid-related indices among all participants. It demonstrated that elevated baseline of BMI, WC, VAI, LAP, TyG, and TyHGB were significantly correlated with an increased risk of T2D events (all *log-rank tests, P* < 0.001). Furthermore, among participants with normal FBG level at baseline (**Supplementary File**: **Supplementary Figure 1**) as well as those exhibiting elevated FBG levels (**Supplementary File**: **Supplementary Figure 2**), statistically significant differences in T2D event risk were observed across quartiles of VAI, LAP, TyG, and TyHGB (*log-rank tests, P* < 0.05).

Cox proportional hazards regression analyses were conducted to examine the associations between obesity- and lipid- indices and the risk of T2D events (**Table 3**). After adjustment for covariates, Model 3 revealed that, relative to the lowest quartile, participants in the highest quartile of baseline BMI, WC, VAI, LAP, TyG, and TyHGB exhibited fully adjusted *HRs* for T2D events of 1.45 (95% *CI*: 1.24–1.70), 1.62 (95% *CI*: 1.39–1.90), 2.35 (95% *CI*: 1.99–2.77), 2.41 (95% *CI*: 2.03–2.85), 4.88 (95% *CI*: 4.04–5.90), and 6.75 (95% *CI*: 5.40–8.45), respectively. When these six indicators were analyzed as continuous variables, Model 3 indicated that each one-standard-deviation increment in BMI, WC, VAI, LAP, TyG, and TyHGB was associated with a corresponding increase in T2D risk, with adjusted *HRs* of 1.17 (95% *CI*: 1.11–1.23), 1.23 (95% *CI*: 1.16–1.30), 1.30 (95% *CI*: 1.24–1.37), 1.21 (95% *CI*: 1.17–1.26), 1.76 (95% *CI*: 1.67–1.86), and 1.37 (95% *CI*: 1.33–1.42), respectively. Additionally, among individuals with either normal FBG or elevated FBG levels (**Supplementary File**: **Supplementary Figures 3**, **4**), those in the highest quartile for VAI, LAP, TyG, and TyHGB demonstrated a significantly greater risk of T2D events compared to those in the lowest quartile (all *P* < 0.05).

**Table 3:** Longitudinal association between novel obesity- and lipid-related indices and onset of T2D among all participants.

Indices	T2D events (%)	HRs (95% *CI*) of T2D					
		Model 1	*P value*	Model 2	*P value*	Model 3	*P value*
BMI (Per 1SD increase)	1350 (7.40)	1.25 (1.19,1.31)	<0.001	1.25 (1.19,1.31)	<0.001	1.17 (1.11,1.23)	<0.001
BMI quartiles							
Q1 (<21.875)	254 (5.59)	*reference*		*reference*		*reference*	
Q2 (21.875 to<23.837)	317 (6.91)	1.25 (1.06,1.47)	0.009	1.25 (1.06,1.48)	0.008	1.17 (0.99,1.38)	0.059
Q3 (23.837 to<25.965)	357 (7.82)	1.42 (1.20,1.66)	<0.001	1.43 (1.22,1.68)	<0.001	1.28 (1.09,1.50)	0.003
Q4 (≥25.965)	422 (9.26)	1.69 (1.45,1.98)	<0.001	1.70 (1.45,1.98)	<0.001	1.45 (1.24,1.70)	<0.001
WC (Per 1SD increase)	1350 (7.40)	1.30 (1.23,1.37)	<0.001	1.31 (1.24,1.38)	<0.001	1.23 (1.16,1.30)	<0.001
WC quartiles							
Q1 (<81.000)	247 (5.52)	*reference*		*reference*		*reference*	
Q2 (81.000 to<86.000)	271 (6.85)	1.25 (1.05,1.49)	0.011	1.25 (1.05,1.49)	0.011	1.17 (0.98,1.39)	0.081
Q3 (86.000 to<92.000)	351 (7.06)	1.29 (1.10,1.52)	0.002	1.30 (1.11,1.53)	0.002	1.18 (1.00,1.39)	0.049
Q4 (≥92.000)	481 (9.91)	1.84 (1.58,2.15)	<0.001	1.87 (1.60,2.18)	<0.001	1.62 (1.39,1.90)	<0.001
VAI (Per 1SD increase)	1350 (7.40)	1.34 (1.28,1.41)	<0.001	1.34 (1.28,1.41)	<0.001	1.30 (1.24,1.37)	<0.001
VAI quartiles							
Q1 (<1.316)	196 (4.30)	*reference*		*reference*		*reference*	
Q2 (1.316 to<2.006)	308 (6.75)	1.56 (1.33,1.90)	<0.001	1.58 (1.32,1.89)	<0.001	1.48 (1.24,1.77)	<0.001
Q3 (2.006 to<3.067)	352 (7.71)	1.82 (1.53,2.17)	<0.001	1.81 (1.52,2.15)	<0.001	1.65 (1.38,1.97)	<0.001
Q4 (≥3.067)	494 (10.83)	2.61 (2.21,3.08)	<0.001	2.59 (2.19,3.05)	<0.001	2.35 (1.99,2.77)	<0.001
LAP (Per 1SD increase)	1350 (7.40)	1.22 (1.18,1.26)	<0.001	1.22 (1.18,1.27)	<0.001	1.21 (1.17,1.26)	<0.001
LAP quartiles							
Q1 (<21.000)	198 (4.36)	*reference*		*reference*		*reference*	
Q2 (21.000to<33.750)	303 (6.61)	1.53 (1.28,1.83)	<0.001	1.52 (1.27,1.81)	<0.001	1.42 (1.19,1.70)	<0.001
Q3 (33.750 to<52.520)	356 (7.80)	1.82 (1.53,2.17)	<0.001	1.81 (1.52,2.15)	<0.001	1.70 (1.43,2.03)	<0.001
Q4 (≥52.520)	493 (10.80)	2.57 (2.18,3.03)	<0.001	2.55 (2.16,3.01)	<0.001	2.41 (2.03,2.85)	<0.001
TyG (Per 1SD increase)	1350 (7.40)	1.68 (1.60,1.76)	<0.001	1.69 (1.60,1.77)	<0.001	1.76 (1.67,1.86)	<0.001
TyG quartiles							
Q1 (<8.313)	139 (3.04)	*reference*		*reference*		*reference*	
Q2 (8.313 to<8.660)	249 (5.47)	1.82 (1.48,2.23)	<0.001	1.82 (1.48,2.24)	<0.001	1.83 (1.48,2.25)	<0.001
Q3 (8.660 to<9.025)	349 (7.66)	2.57 (2.11,3.13)	<0.001	2.59 (2.13,3.16)	<0.001	2.62 (2.15,3.20)	<0.001
Q4 (≥9.025)	613 (13.43)	4.67 (3.88,5.61)	<0.001	4.65 (3.87,5.59)	<0.001	4.88 (4.04,5.90)	<0.001
TyHGB (Per 1SD increase)	1350 (7.40)	1.36 (1.33,1.41)	<0.001	1.37 (1.33,1.41)	<0.001	1.37 (1.33,1.42)	<0.001
TyHGB quartiles							
Q1 (<7.582)	89 (1.95)	*reference*		*reference*		*reference*	
Q2 (7.582 to<8.641)	225 (4.93)	2.57 (2.01,3.28)	<0.001	2.58 (2.02,3.30)	<0.001	2.40 (1.87,3.07)	<0.001
Q3 (8.641 to<10.031)	412 (9.03)	4.79 (3.81,6.02)	<0.001	4.79 (3.81,6.03)	<0.001	4.40 (3.49,5.54)	<0.001
Q4 (≥10.031)	624 (13.67)	7.47 (5.98,9.32)	<0.001	7.42 (5.94,9.27)	<0.001	6.75 (5.40,8.45)	<0.001

Model 1, no confounders were included; model 2, age, gender, educational level, smoking, were included. Model 3, Hb, hypertension, SBP, DBP and TC based on Model 2.

T2D, type 2 diabetes; HRs, hazard ratios; SD, Standard deviation; BMI, body math index; WC, waist circumference; VAI, visceral adiposity index; LAP, lipid accumulation product; TyG, triglyceride glucose index; TyHGB, triglyceride high-density cholesterol-glucose body index.

### Dose-response relationship between obesity- and novel- indices and T2D

**Figure 3:**
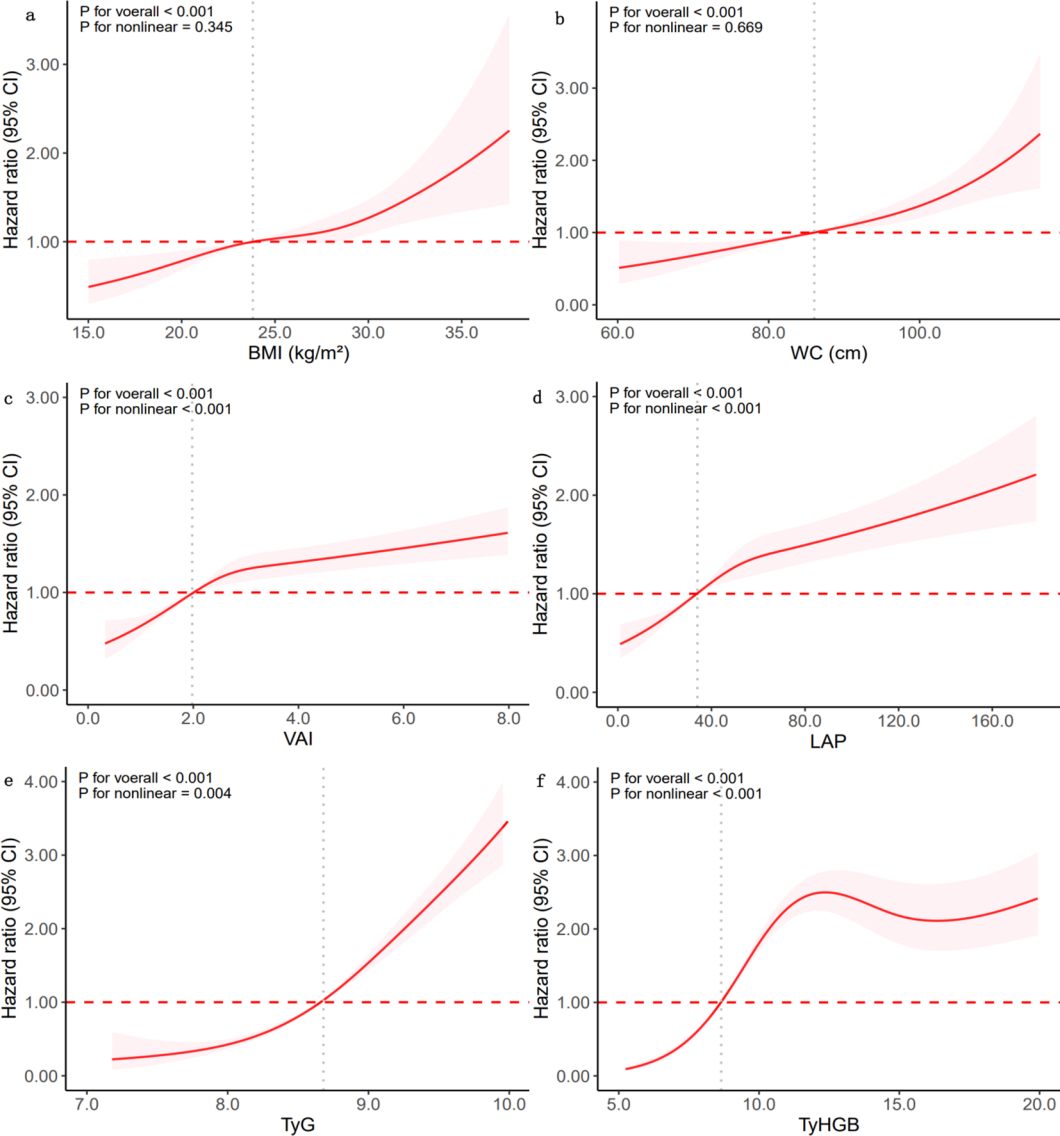
The nonlinear relationship between novel obesity- and lipid-related indices and T2D among all participants. Adjustment factors included age, gender, education, marital status, smoking, alcohol consumption, hypertension, diabetic family history, SBP, DBP, Hb, TC, and LDL-C. **(a)** BMI, **(b)** WC, **(c)** VAI, **(d)** LAP, **(e)** TyG, **(f)** TyHGB.

After adjusting for confounders, a nonlinear association between the novel obesity-and lipids- indices and the onset of T2D among all participants was examined via a *GAM* based on a *RCS* (**Figure 3**). The results indicated that BMI and WC have linear associations with T2D risk in the overall population (*p* for nonlinear > 0.05), whereas VAI, LAP, TyG, and TyHGB exhibited nonlinear associations (*p* for nonlinear< 0.05). In the normal FBG group (**Supplementary File**: **Supplementary Figure 5**), TyG showed a linear relationship with T2D risk (*p* for nonlinear = 0.094), while BMI, VAI, LAP, and TyHGB displayed nonlinear relationships (*p* for nonlinear< 0.05). WC, VAI, LAP, and TyG were linearly related with the onset of T2D in elevated FBG group (*p* for nonlinear > 0.05)(**Supplementary File**: **Supplementary Figure 6**), whereas TyHGB demonstrated a nonlinear association with T2D risk (*p* for nonlinear< 0.05).

### Predictive performance of obesity- and lipid- indices for T2D

**Figure 4:**
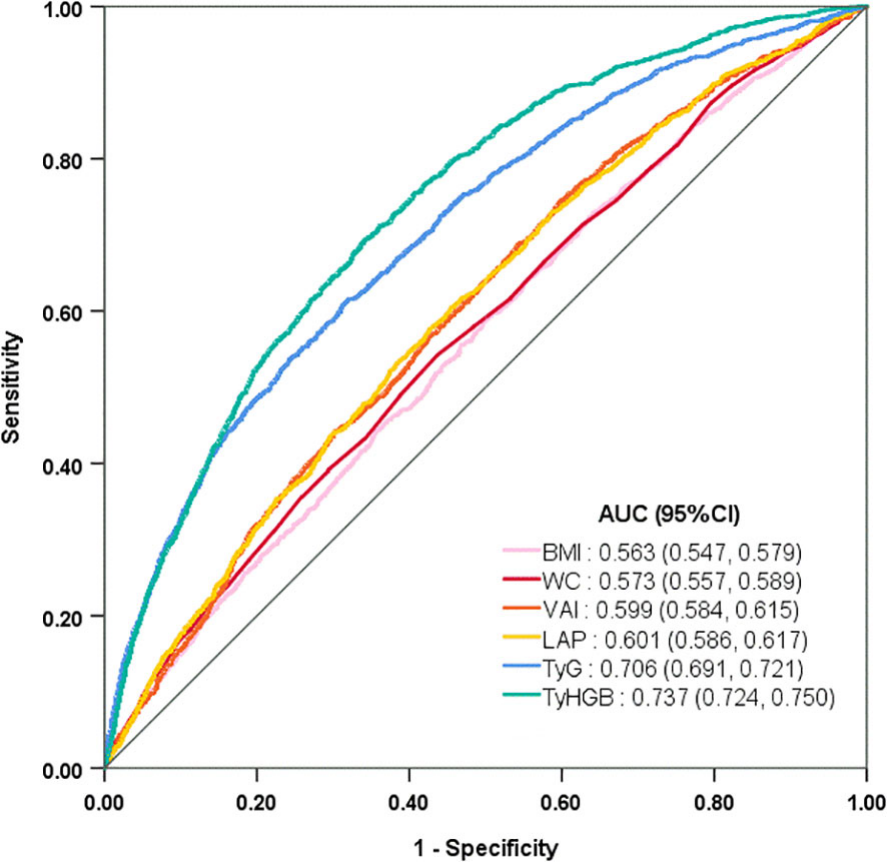
The ROC curves for novel obesity- and lipid-related indices in prediction of T2D among all participants.

To assess the predictive capabilities of various obesity- and lipid-related indices for the onset of T2D, *ROC* analysis was performed (**Table 4**). **Figure 4** illustrated the *ROC* curves for each index predicting T2D in all participants. The findings revealed that, among all participants, the TyHGB (*AUC* = 0.737, 95% *CI*: 0.724–0.750) and TyG (*AUC* = 0.706, 95% *CI*: 0.691–0.721) exhibited relatively strong predictive efficacy for T2D, These were followed by VAI (*AUC* = 0.599, 95% *CI*: 0.584–0.615) and LAP (*AUC* = 0.601, 95% *CI*: 0.586–0.617). In contrast, BMI (*AUC* = 0.563, 95% *CI*: 0.548–0.579) and WC (*AUC* = 0.573, 95% *CI*: 0.557–0.589) demonstrated the lowest predictive performance. With the exception of BMI, the other five indices showed statistically significant differences between the normal FBG group (**Supplementary File**: **Supplementary Figure 7**) and the elevated FBG group (**Supplementary File**: **Supplementary Figure 8**) (*P<* 0.05). Furthermore, TyG and TyHGB demonstrated superior predictive performance compared to the other four indices, as detailed in **Table 4**.

**Table 4:** Predictive performance of novel obesity- and lipid-related indices for T2D.

Indices	AUC (95%CI)	*P value*	Optional cutoffs	J-youden	Sensitivity	Specificity	(+)LR	(-)LR
All participants								
BMI	0.563 (0.548,0.579)	<0.001	22.97	0.085	0.697	0.388	1.139	0.781
WC	0.573 (0.557,0.589)	<0.001	87.25	0.105	0.542	0.563	1.240	0.813
VAI	0.599 (0.584,0.615)	<0.001	1.70	0.146	0.744	0.402	1.244	0.637
LAP	0.601 (0.586,0.617)	<0.001	37.60	0.150	0.576	0.574	1.352	0.739
TyG	0.706 (0.691,0.721)	<0.001	8.89	0.296	0.616	0.68	1.925	0.565
TyHGB	0.737 (0.724,0.750)	<0.001	9.34	0.352	0.694	0.658	2.029	0.465
Normal FBG participants								
BMI	0.517 (0.487,0.547)	0.252	27.21	0.000	0.125	0.875	1.000	1.000
WC	0.531 (0.502,0560)	0.042	77.65	0.064	0.904	0.16	1.076	0.600
VAI	0.557 (0.528,0.585)	<0.001	1.67	0.115	0.699	0.416	1.197	0.724
LAP	0.550 (0.522,0.577)	0.001	21.76	0.112	0.816	0.296	1.159	0.622
TyG	0.568 (0.540,0.597)	<0.001	8.41	0.116	0.733	0.383	1.188	0.697
TyHGB	0.588 (0.561,0.615)	<0.001	7.65	0.126	0.787	0.339	1.191	0.628
Elevated FBG participants								
BMI	0.519 (0.499,0.540)	0.069	23.07	0.033	0.722	0.311	1.048	0.894
WC	0.535 (0.514,0.556)	0.001	90.25	0.056	0.423	0.633	1.153	0.912
VAI	0.551 (0.530,0572)	<0.001	2.69	0.088	0.484	0.604	1.222	0.854
LAP	0.544 (0.523,0.565)	<0.001	55.09	0.084	0.393	0.691	1.272	0.878
TyG	0.624 (0.603,0.644)	<0.001	9.24	0.204	0.513	0.691	1.660	0.705
TyHGB	0.661 (0.641,0.680)	<0.001	10.28	0.248	0.667	0.581	1.592	0.573

### Subgroup analysis and sensitivity analysis

**Figure 5:**
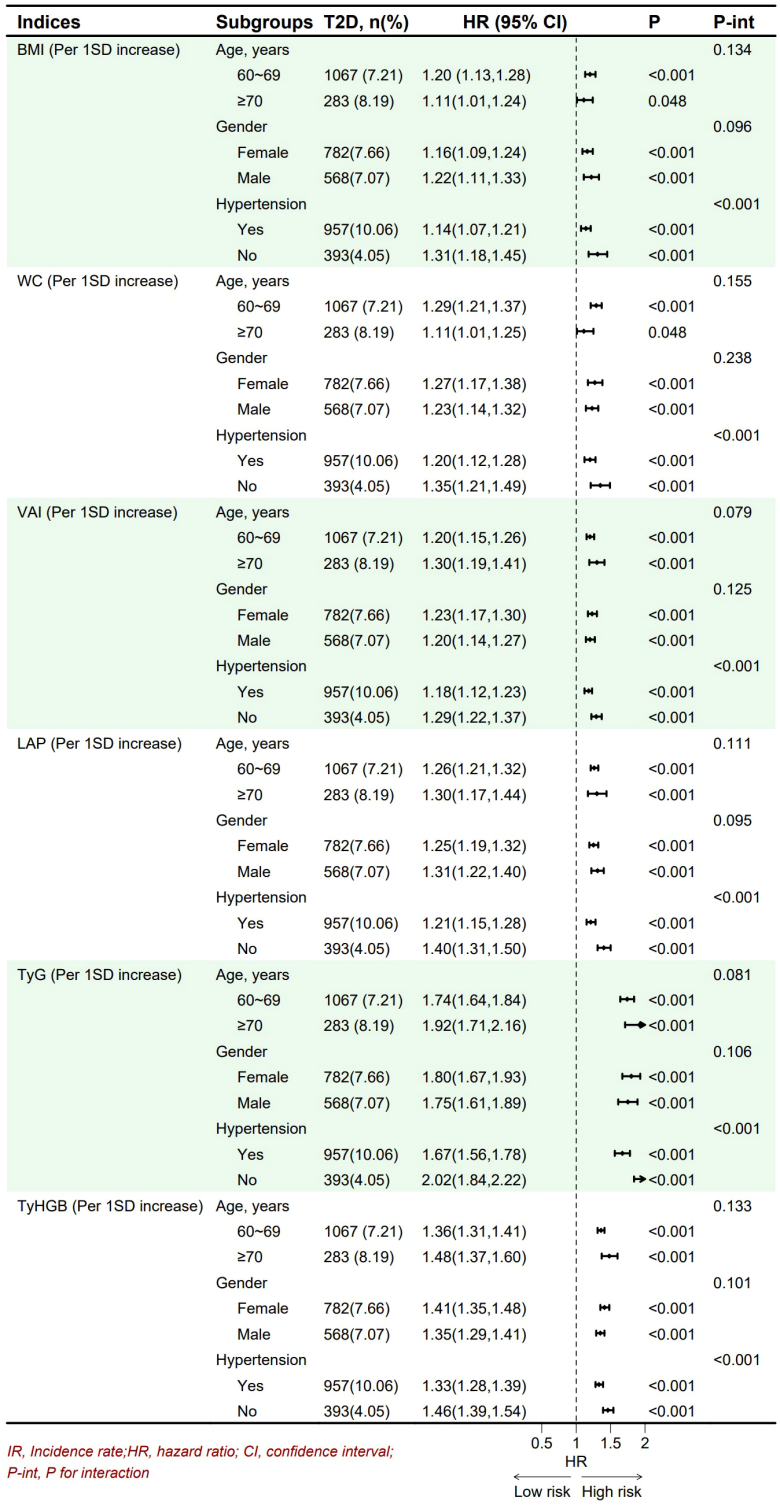
Association between novel obesity- and lipid-related indices and onset of T2D in different subgroups.

To assess the generalizability of each indices across different populations, subgroup analyses were performed stratified by age (60–69 years and ≥70 years), gender (female and male), and hypertension status (yes or no). The findings indicated that the associations between the indices and the risk of T2D persisted within these subgroups after adjusting potential confounders (**Figure 5**). Notably, a significant interaction effect was identified between age and hypertension status (all *p* < 0.05), revealing that the indices exerted a more pronounced influence on T2D risk among individuals without hypertension. No significant interactions were detected for the other indices (all *p* > 0.05) (**Figure 5**). To confirm the robustness of these results, analyses were repeated after excluding 152 participants who died during the follow-up period. The associations between the novel obesity- and lipid-related indices and new onset of T2D persisted (**Supplementary File**: **Supplementary Table 1**).

## Discussion

This study, based on a large elderly cohort dataset, assessed the predictive capacity of six obesity- and lipid-related indices for the risk of T2D in the elderly population across different FBG levels. After adjusting for confounding factors, the analysis revealed that all six indices were significantly associated with the onset T2D in the elderly, consistent with findings from previous studies (Feng, 2023; Wang, 2023; Brahimaj, 2019). Specifically, among elderly individuals with normal FBG, only VAI, LAP, TyG, and TyHGB demonstrated positive correlations with onset T2D. Conversely, in participants with elevated FBG, WC, VAI, LAP, TyG, and TyHGB were positively associated with the onset T2D. This positive association persisted across different subgroups, including age, sex, and hypertension status. RCS analysis further indicated that the risk of developingT2D increases progressively with higher levels of obesity- and lipid-related indices, underscoring their significant predictive value in the elderly populations.

T2D has emerged as a significant global public health challenge. Early identification of individuals at risk for diabetes is essential for effective prevention and management, as it can mitigate disease onset and reduce the strain on healthcare systems. Consequently, there is an urgent demand for a simple, cost-effective, and practical biomarker to predict the likelihood of developing T2D. Obesity is a well-established risk factor for T2D; however, conventional measures such as BMI and WC offer only a general assessment of obesity and fail to capture fat distribution patterns (34). Prior research suggests that alternative indices reflecting abdominal fat accumulation provide superior predictive accuracy for CVD compared to BMI in the general population (17, 32), though their efficacy in forecasting T2D risk among elderly individuals remains uncertain. Our study revealed that among six indices, TyG, TyHGB, VAI, and LAP demonstrate enhanced predictive performance for T2D relative to traditional metrics such as BMI and WC. This observation is consistent with findings from previous investigations (16, 23), likely attributable to the fact that these four indices integrate parameters including BMI, waist circumference, lipid profiles, and blood glucose levels, thereby surpassing the predictive capacity of single-factor measures. Nonetheless, some studies have reported inconsistent results regarding the predictive validity of VAI, LAP, TyG, and TyHGB (22, 35). Our findings indicate that TyG and TyHGB exhibit superior predictive ability for the onset of T2D compared to VAI and LAP. This discrepancy may be explained by variations in the demographic characteristics of participants or by the inclusion of FBG measurements in TyG and TyHGB, in addition to lipid parameters.

Previous retrospective studies have demonstrated that BMI and WC are closely associated with the incidence of onset of T2D (36, 37). The current study corroborates that both BMI and WC independently correlate with the development of T2D among elderly populations. Nevertheless, this association was not statistically significant in individuals exhibiting either normal or elevated FBG levels. Furthermore, the predictive capacities of BMI (AUC = 0.563, 95% CI: 0.548–0.579) and WC (AUC = 0.573, 95% CI: 0.557–0.589) for onset T2D were relatively modest. This limited predictive power may be attributed to BMI inability to differentiate between muscle and fat mass, and WC representation of total abdominal fat without adequately capturing fat distribution nuances (34). In contrast, VAI, which integrates WC, TG, and HDL indicators, and LAP combining WC and TG indicators, more effectively reflect abdominal lipid accumulation. Within this study, these indices exhibited marginally superior predictive performance for onset T2D in elderly individuals compared to BMI and WC. However, findings from other regional cohorts have been inconsistent (38, 39), potentially due to heterogeneity in study populations and regional variations in fat distribution patterns (40). Additionally, some literature suggests that although VAI and LAP capture aspects of abdominal lipid deposition and associated toxicity, they remain limited in distinguishing visceral adipose tissue from subcutaneous fat compartments (41, 42). The study further revealed that VAI (AUC = 0.599, 95% CI: 0.584–0.615) and LAP (AUC = 0.601, 95% CI: 0.586–0.617) demonstrated weaker predictive efficacy for onset T2D in the elderly compared to TyG (AUC = 0.706, 95% CI: 0.691–0.721) and TyHGB (AUC = 0.737, 95% CI: 0.724–0.750). It is well established that IR and pancreatic β-cell dysfunction constitute the principal pathophysiological mechanisms underlying T2D (43, 44). Adipose tissue secretes numerous hormones and cytokines that exert significant regulatory effects on glucose and lipid metabolism (45). IR is a common pathological feature across various metabolic disorders, including hyperglycemia and hypertriglyceridemia (46). Ahn et al. have demonstrated that the TyG index, which integrates lipid and glucose parameters, reliably reflects insulin resistance in humans (47). Multiple studies conducted within Asian populations have reported a significant association between the TyG index and the risk of developing T2D (48–50). The present study extends these findings by demonstrating that, among elderly cohorts—including both normal FBG and elevated FBG groups—the TyG index exhibits superior predictive efficacy for T2D onset compared to BMI, WC, VAI, and LAP. The TyHGB index, which incorporates key parameters such as TyG (TG and FBG), HDL-C, and pre-pregnancy BMI, was also validated as an effective predictor of T2D in this population. Both TyG and TyHGB indices are cost-effective, readily accessible, and straightforward to compute, rendering them highly applicable for clinical diagnostics and large-scale epidemiological investigations. Consequently, these indices hold considerable promise for the early identification and screening of populations at elevated risk for T2D (51).

T2D is a chronic disease condition for which early identification, coupled with the maintenance of a healthy lifestyle is essential for effective prevention. This study revealed the association between obesity- and lipid-related indices, and the incidence of onset T2D among the elderly population. The research contributes to the early identification of T2D, facilitating timely interventions through lifestyle modifications—such as balanced nutrition and moderate physical activity—to delay or prevent T2D development. The results offer valuable insights for clinical practice, health counseling, and the screening of populations at elevated risk.

Utilizing extensive longitudinal data, this study represents the first to assess the longitudinal associations between novel obesity- and lipid- related indices and the onset of T2D in elderly individuals across different FBG levels. The findings indicate causal relationships and are representative of the studied population. Furthermore, prior research has provided limited evidence concerning the association between TyHGB and the onset of T2D; this study substantially enhances the existing literature in this area. Importantly, the operational definition of T2D events in the study encompasses not only self-reported diagnosis histories but also objective biological markers, including FBG, random blood glucose, and HAb1c levels, thereby reducing the likelihood of underdiagnosis. The measurement techniques employed for the indices are straightforward and readily implementable in clinical practice. All data collection was conducted through one-on-one interviews by trained professionals using structured questionnaires, while physical examinations and laboratory assessments were performed by medical personnel, ensuring the consistency and reliability of the data obtained.

Notwithstanding the strengths of the present study, several limitations merit careful consideration. First, although adjustments were made for a variety of potential confounding variables informed by the study, certain possible confounders—such as physical activity levels and dietary intake—were excluded from the analysis due to limitations in data availability. Second, some participant characteristics were derived from self-reported questionnaire data, which may be susceptible to recall bias. Third, the homogeneity of the study population limits the generalizability of these findings. VAI was originally developed based on European cohorts, and its applicability across other ethnic groups, geographic regions, or healthcare settings remains insufficiently established. Future research should consider developing population-specific calibration strategies, including establishing race-specific thresholds, and explore integrating additional anthropometric measurements or metabolic markers to enhance predictive performance across populations. Furthermore, prospective validation studies across multi-ethnic cohorts, rural populations, and diverse healthcare systems are needed to confirm the robustness of VAI as a universal indicator of visceral fat mass and cardiovascular metabolic risk. Additionally, the study predicts the risk of developingT2D within two years based solely on a single baseline measurement of indicators and covariates, despite the fact that these variables may change over time. We will continue to refine follow-up data and employ time-dependent Cox models or repeated measures analysis to validate the causal relationship between each indicator and T2D.

## Conclusion

This Longitudinal study suggested a positive correlation between most of novel obesity- and lipid-related indices and new onset of T2D among the elderly, providing new evidence for a causal relation. TyG and TyHGB exhibit the highest predictive efficacy for the onset of T2D in the elderly population, making them essential for the implementation of early screening programs targeting this demographic.

## Data Availability

The original contributions presented in the study are included in the article/**Supplementary Material**. Further inquiries can be directed to the corresponding author.

## Supplementary material

Supplementary Figure 1 Kaplan - Meier incidence rate of T2D according to quartiles of novel obesity- and lipid-related indices among normal FBG participants. **(a)** BMI, **(b)** WC, **(c)** VAI, **(d)** LAP, **(e)** TyG, **(f)**, TyHGB.

Supplementary Figure 2 Kaplan - Meier incidence rate of T2D according to quartiles of novel obesity- and lipid-related indices among elevated FBG participants. **(a)** BMI, **(b)** WC, **(c)** VAI, **(d)** LAP, **(e)** TyG, **(f)**, TyHGB.

Supplementary Figure 3 Forest diagram of relationship between novel obesity- and lipid-related indices and T2D among normal FBG participants.

Supplementary Figure 4 Forest diagram of relationship between novel obesity- and lipid-related indices and T2D among elevated FBG participants

Supplementary Figure 5 The nonlinear relationship between novel obesity- and lipid-related indices and T2D among normal FBG participants.

Supplementary Figure 6 The nonlinear relationship between novel obesity- and lipid-related indices and T2D among elevated FBG participants.

Supplementary Figure 7 The ROC curves for novel obesity- and lipid-related indices in prediction of T2D among normal FBG participants.

Supplementary Figure 8 The ROC curves for novel obesity- and lipid-related indices in prediction of T2D among elevated FBG participants.
