# Supplementary material for: Association between novel obesity- and lipid-related indices and diabetes across different FBG among elderly: a prospective cohort study
Source: Front Endocrinol (Lausanne). 2026 Mar 26;17:1798687. doi: 10.3389/fendo.2026.1798687 (PMC13063830; doi:10.3389/fendo.2026.1798687)
Supplement: Supplementary Figure 1 — Kaplan - Meier incidence rate of T2D according to quartiles of novel obesity- and lipid-related indices among normal FBG participants. (a) BMI, (b) WC, (c) VAI, (d) LAP, (e) TyG, (f), TyHGB. [file DataSheet1.zip › Table S1.docx]

**Table S1**. Longitudinal association between novel obesity- and lipid-related indices and new-oneset of T2D among all participants

| Indices | T2D events (%) | HRs (95% *CI*) of T2D | | | | | |
| --- | --- | --- | --- | --- | --- | --- | --- |
|  |  | Model1 | *P value* | Model2 | *P value* | Model3 | *P value* |
| BMI (Per 1SD increase) | 1335(7.36) | 1.26(1.19,1.32) | <0.001 | 1.26(1.19,1.32) | <0.001 | 1.17(1.11,1.24) | <0.001 |
| BMI quartiles |  |  |  |  |  |  |  |
| Q1 (<21.879) | 246(5.47) | *reference* |  | *reference* |  | *reference* |  |
| Q2 (21.875 to <23.850) | 313(6.85) | 1.26(1.07,1.49) | 0.009 | 1.26(1.06,1.49) | 0.007 | 1.19(1.00,1.39) | 0.055 |
| Q3 (23.837 to <25.970) | 355(7.78) | 1.44(1.22,1.69) | <0.001 | 1.43(1.22,1.69) | <0.001 | 1.29(1.09,1.52) | 0.003 |
| Q4 (≥25.970) | 421(9.34) | 1.75(1.49,2.05) | <0.001 | 1.72(1.47,2.02) | <0.001 | 1.49(1.27,1.74) | <0.001 |
| WC (Per 1SD increase) | 1335(7.36) | 1.31(1.24,1.38) | <0.001 | 1.31(1.25,1.38) | <0.001 | 1.24(1.17,1.31) | <0.001 |
| WC quartiles |  |  |  |  |  |  |  |
| Q1 (<81.000) | 244(5.50) | *reference* |  | *reference* |  | *reference* |  |
| Q2 (81.000 to <86.000) | 263(6.69) | 1.23(1.03,1.46) | 0.009 | 1.22(1.03,1.46) | 0.008 | 1.14(0.96,1.36) | 0.142 |
| Q3 (86.000 to <92.000) | 350(7.07) | 1.30(1.10,1.53) | <0.001 | 1.31(1.11,1.54) | <0.001 | 1.18(1.00,1.39) | 0.048 |
| Q4 (≥92.000) | 478(9.92) | 1.85(1.59,2.16) | <0.001 | 1.87(1.60,2.19) | <0.001 | 1.62(1.39,1.90) | <0.001 |
| VAI (Per 1SD increase) | 1335(7.36) | 1.22(1.18,1.27) | <0.001 | 1.22(1.18,1.27) | <0.001 | 1.21(1.17,1.26) | <0.001 |
| VAI quartiles |  |  |  |  |  |  |  |
| Q1 (<1.317) | 195(4.30) | *reference* |  | *reference* |  | *reference* |  |
| Q2 (1.316 to <2.007) | 302(6.66) | 1.57(1.31,1.88) | <0.001 | 1.56(1.30,1.86) | <0.001 | 1.45(1.21,1.74) | <0.001 |
| Q3 (2.006 to <3.068) | 349(7.69) | 1.82(1.53,2.17) | <0.001 | 1.80(1.51,2.14) | <0.001 | 1.64(1.37,1.96) | <0.001 |
| Q4 (≥3.068) | 489(10.79) | 2.60(2.20,3.07) | <0.001 | 2.57(2.18,3.04) | <0.001 | 2.33(1.97,2.75) | <0.001 |
| LAP (Per 1SD increase) | 1335(7.36) | 1.26(1.22,1.31) | <0.001 | 1.27(1.22,1.32) | <0.001 | 1.26(1.21,1.32) | <0.001 |
| LAP quartiles |  |  |  |  |  |  |  |
| Q1 (<21.060) | 194(4.29) | *reference* |  | *reference* |  | *reference* |  |
| Q2 (21.000to <33.750) | 297(6.55) | 1.55(1.29,1.85) | <0.001 | 1.53(1.28,1.83) | <0.001 | 1.43(1.20,1.72) | <0.001 |
| Q3 (33.750 to <52.560) | 353(7.78) | 1.85(1.55,2.20) | <0.001 | 1.83(1.53,2.18) | <0.001 | 1.72(1.44,2.06) | <0.001 |
| Q4 (≥52.560) | 491(10.82) | 2.62(2.22,3.09) | <0.001 | 2.59(2.19,3.06) | <0.001 | 2.45(2.06,2.90) | <0.001 |
| TyG (Per 1SD increase) | 1335(7.36) | 1.68(1.60,1.77) | <0.001 | 1.69(1.61,1.77) | <0.001 | 1.77(1.67,1.86) | <0.001 |
| TyG quartiles |  |  |  |  |  |  |  |
| Q1 (<8.314) | 137(3.02) | *reference* |  | *reference* |  | *reference* |  |
| Q2 (8.313 to <8.661) | 244(5.38) | 1.80(1.46,2.20) | <0.001 | 1.79(1.45,2.21) | <0.001 | 1.80(1.46,2.22) | <0.001 |
| Q3 (8.660 to <9.027) | 348(7.68) | 2.60(2.13,3.16) | <0.001 | 2.59(2.13,3.16) | <0.001 | 2.64(2.16,3.22) | <0.001 |
| Q4 (≥9.027) | 606(13.38) | 4.68(3.89,5.63) | <0.001 | 4.63(3.85,5.57) | <0.001 | 4.88(4.04,5.91) | <0.001 |
| TyHGB (Per 1SD increase) | 1335(7.36) | 1.37(1.33,1.41) | <0.001 | 1.37(1.33,1.41) | <0.001 | 1.37(1.33,1.42) | <0.001 |
| TyHGB quartiles |  |  |  |  |  |  |  |
| Q1 (<7.584) | 87(1.92) | *reference* |  | *reference* |  | *reference* |  |
| Q2 (7.582 to <8.642) | 224(4.94) | 2.61(2.04,3.35) | 0.009 | 2.60(2.03,3.33) | 0.008 | 2.43(1.90,3.12) | 0.059 |
| Q3 (8.641 to <10.032) | 406(8.94) | 4.82(3.82,6.08) | <0.001 | 4.78(3.79,6.03) | <0.001 | 4.41(3.50,5.57) | 0.003 |
| Q4 (≥10.032) | 618(13.63) | 7.56(6.04,9.47) | <0.001 | 7.46(5.96,9.34) | <0.001 | 6.82(5.44,8.55) | <0.001 |

Model 1, no confounders were included; model 2, age, gender, educational level, marital status, smoking, alcohol consumption were included. Model 3, Hb, hypertension, SBP, DBP, TC and LDL-C based on Model 2.

T2D, type 2 diabetes; HRs, hazard ratios; SD, Standard deviation; BMI, body math index; WC, waist circumference; VAI, visceral adiposity index; LAP, lipid accumulation product; TyG, triglyceride glucose index; TyHGB, triglyceride high-density cholesterol-glucose body index.
