# Supplementary figures and images for: Association between novel obesity- and lipid-related indices and diabetes across different FBG among elderly: a prospective cohort study
Source: Front Endocrinol (Lausanne). 2026 Mar 26;17:1798687. doi: 10.3389/fendo.2026.1798687 (PMC13063830; doi:10.3389/fendo.2026.1798687)

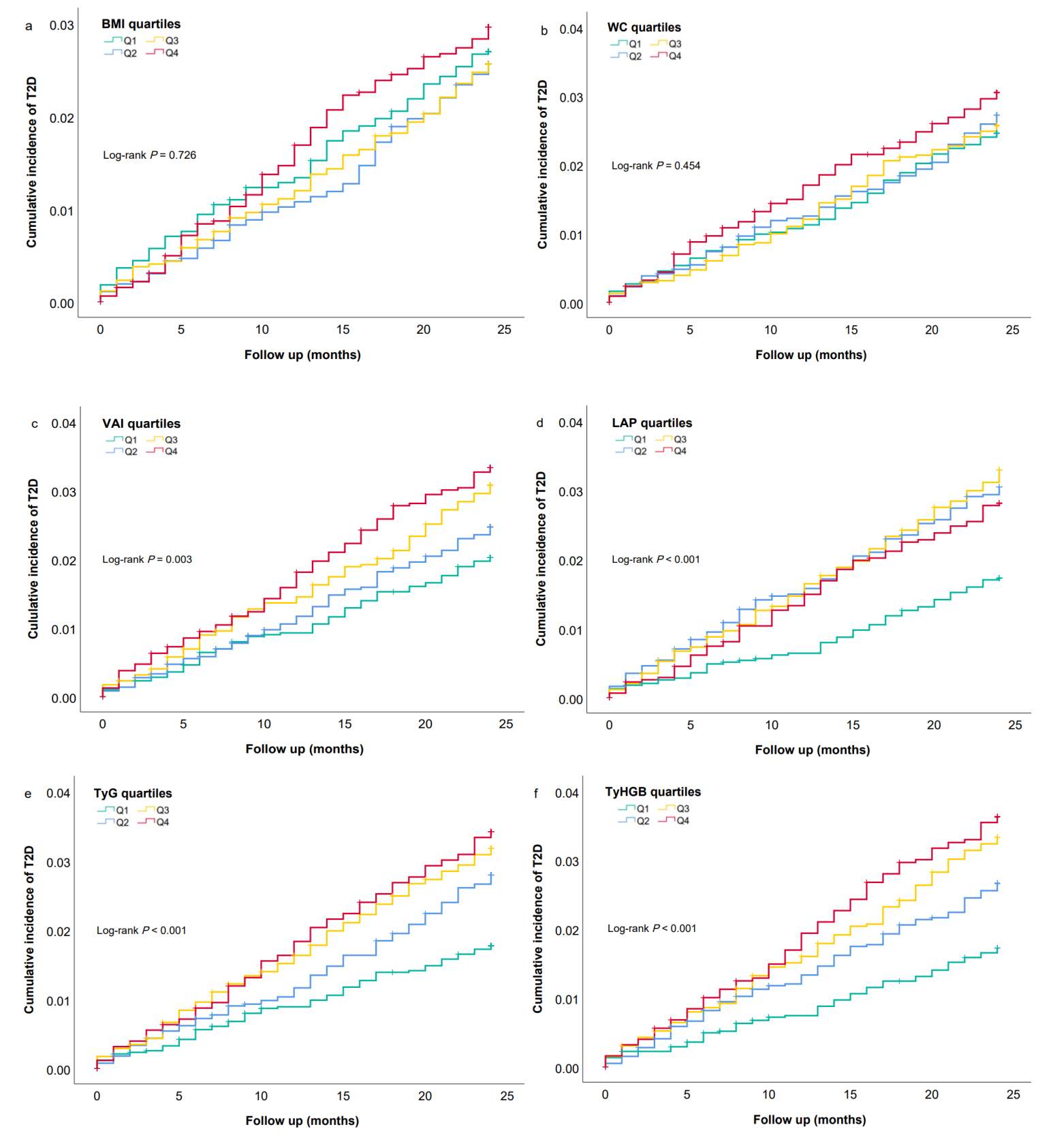

Supplement: Supplementary Figure 1 — Kaplan - Meier incidence rate of T2D according to quartiles of novel obesity- and lipid-related indices among normal FBG participants. (a) BMI, (b) WC, (c) VAI, (d) LAP, (e) TyG, (f), TyHGB. [file DataSheet1.zip › Figure S1.tif]

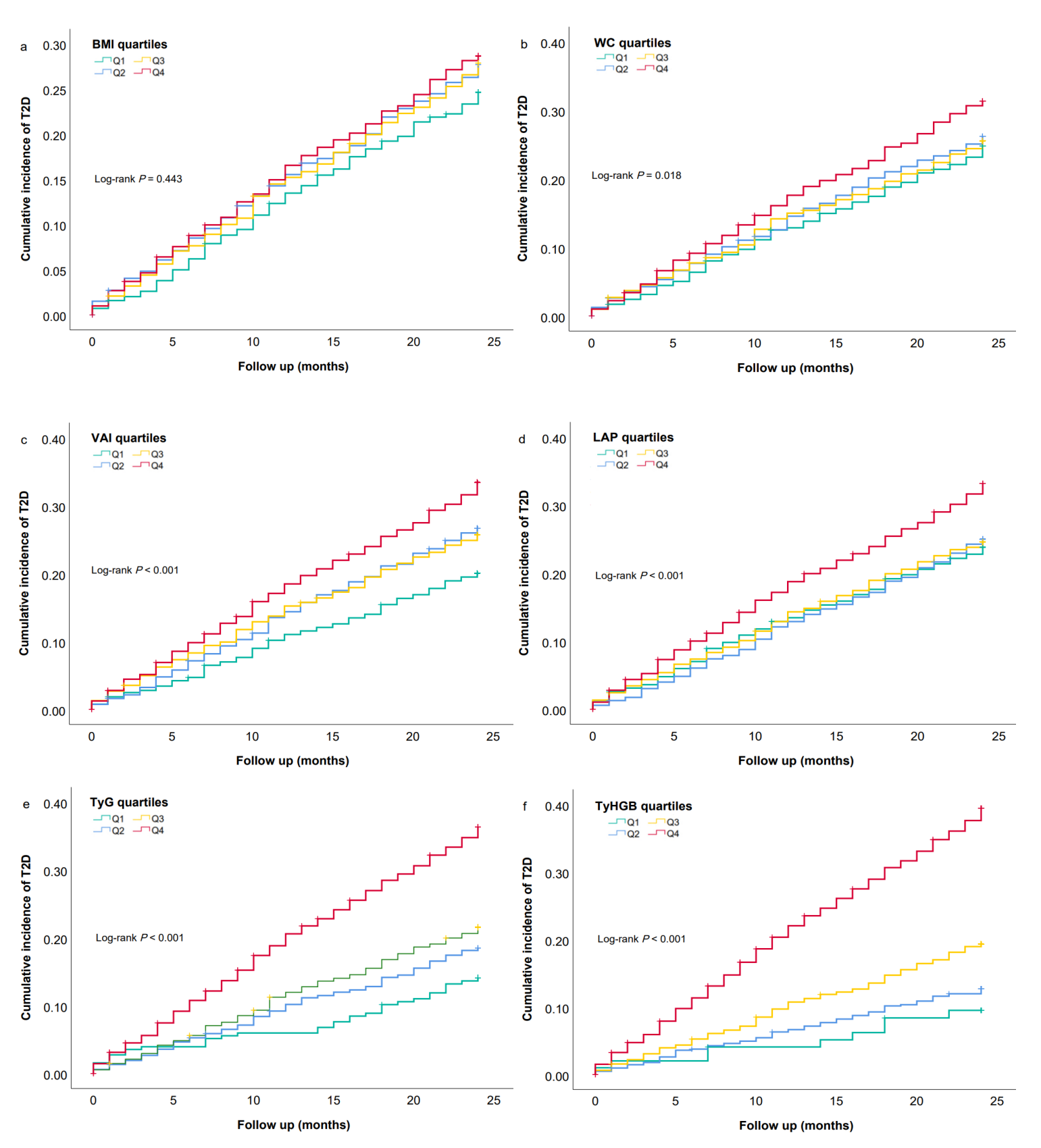

Supplement: Supplementary Figure 1 — Kaplan - Meier incidence rate of T2D according to quartiles of novel obesity- and lipid-related indices among normal FBG participants. (a) BMI, (b) WC, (c) VAI, (d) LAP, (e) TyG, (f), TyHGB. [file DataSheet1.zip › Figure S2.tif]

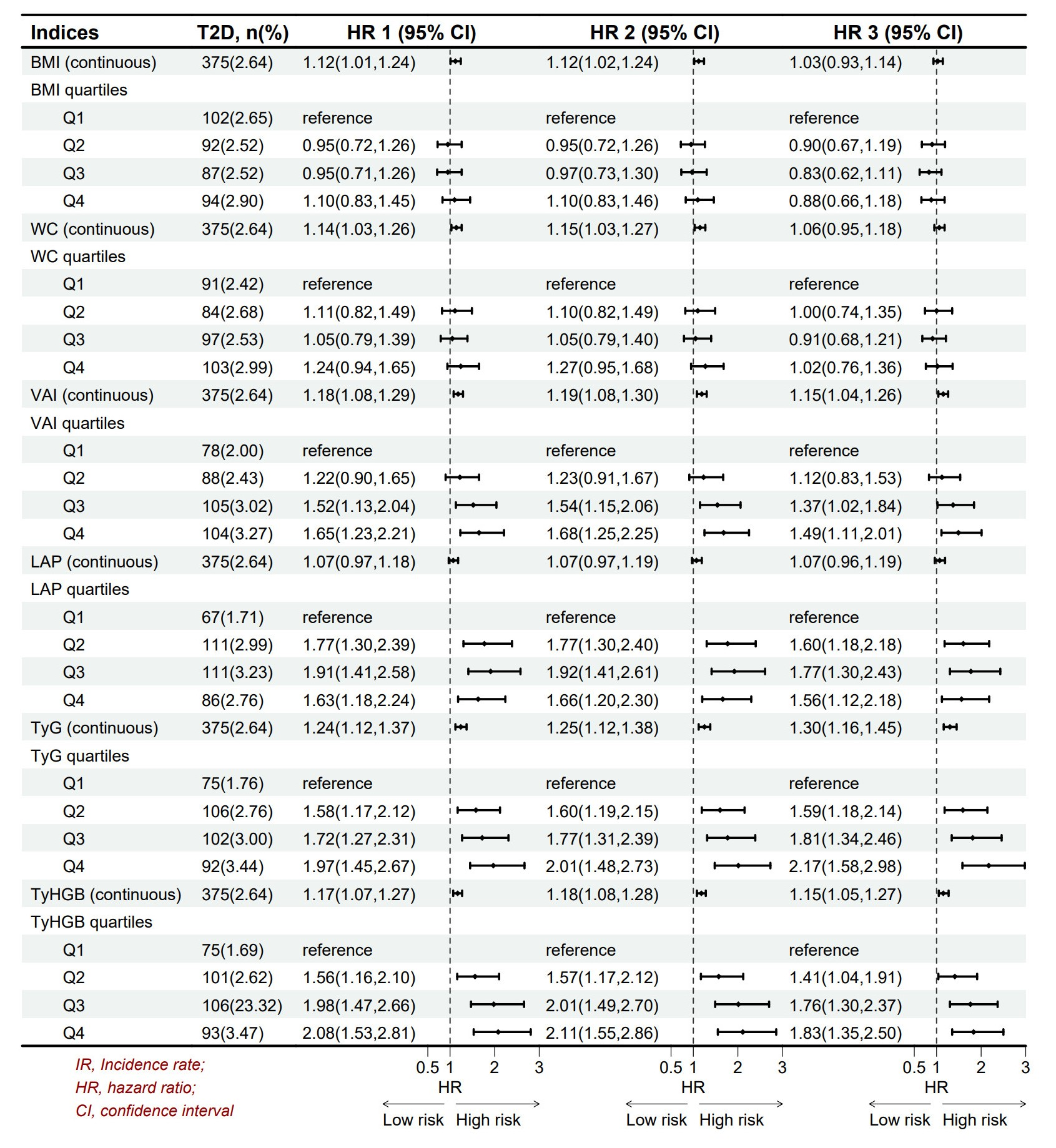

Supplement: Supplementary Figure 1 — Kaplan - Meier incidence rate of T2D according to quartiles of novel obesity- and lipid-related indices among normal FBG participants. (a) BMI, (b) WC, (c) VAI, (d) LAP, (e) TyG, (f), TyHGB. [file DataSheet1.zip › Figure S3.tif]

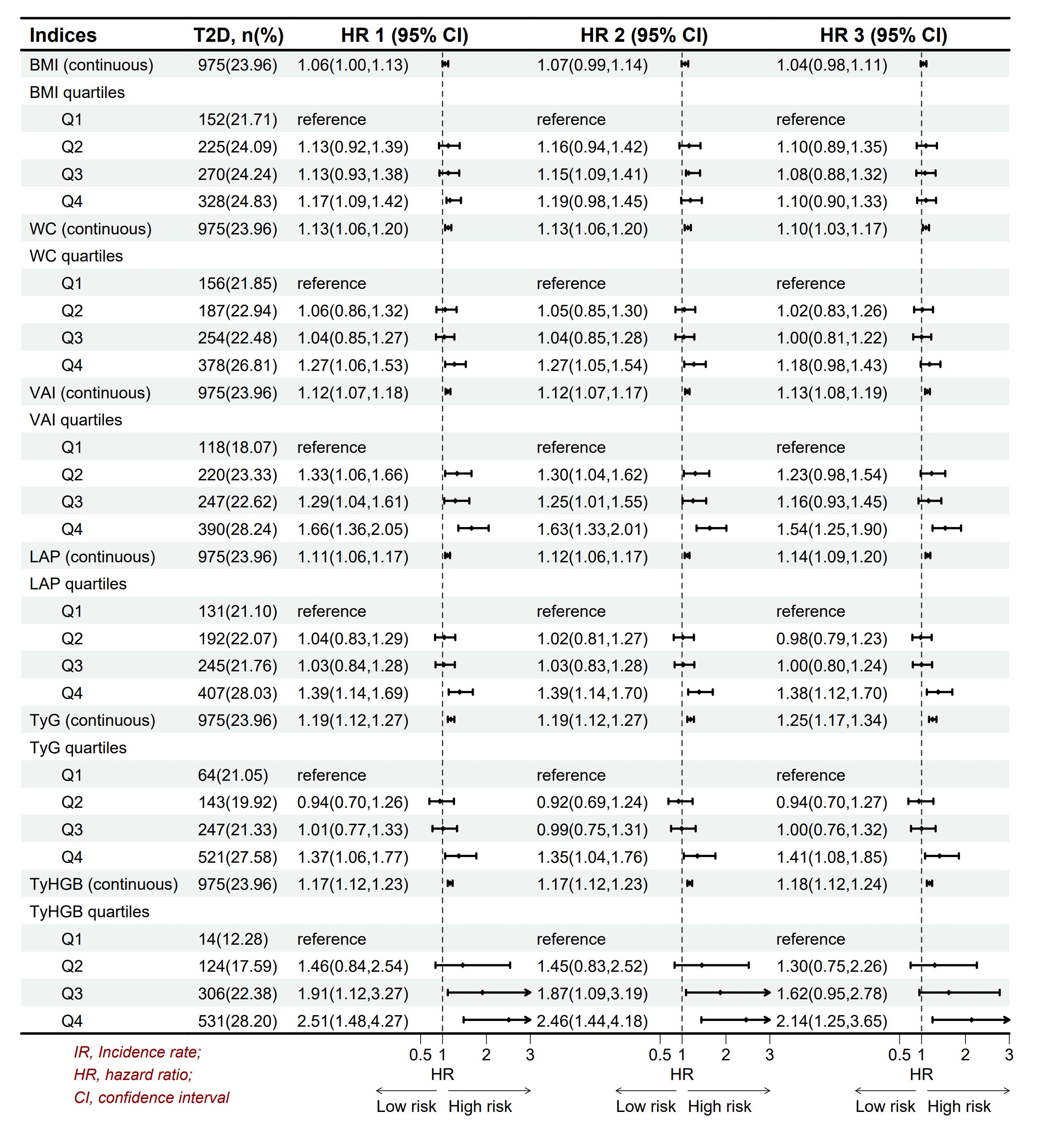

Supplement: Supplementary Figure 1 — Kaplan - Meier incidence rate of T2D according to quartiles of novel obesity- and lipid-related indices among normal FBG participants. (a) BMI, (b) WC, (c) VAI, (d) LAP, (e) TyG, (f), TyHGB. [file DataSheet1.zip › Figure S4.tif]

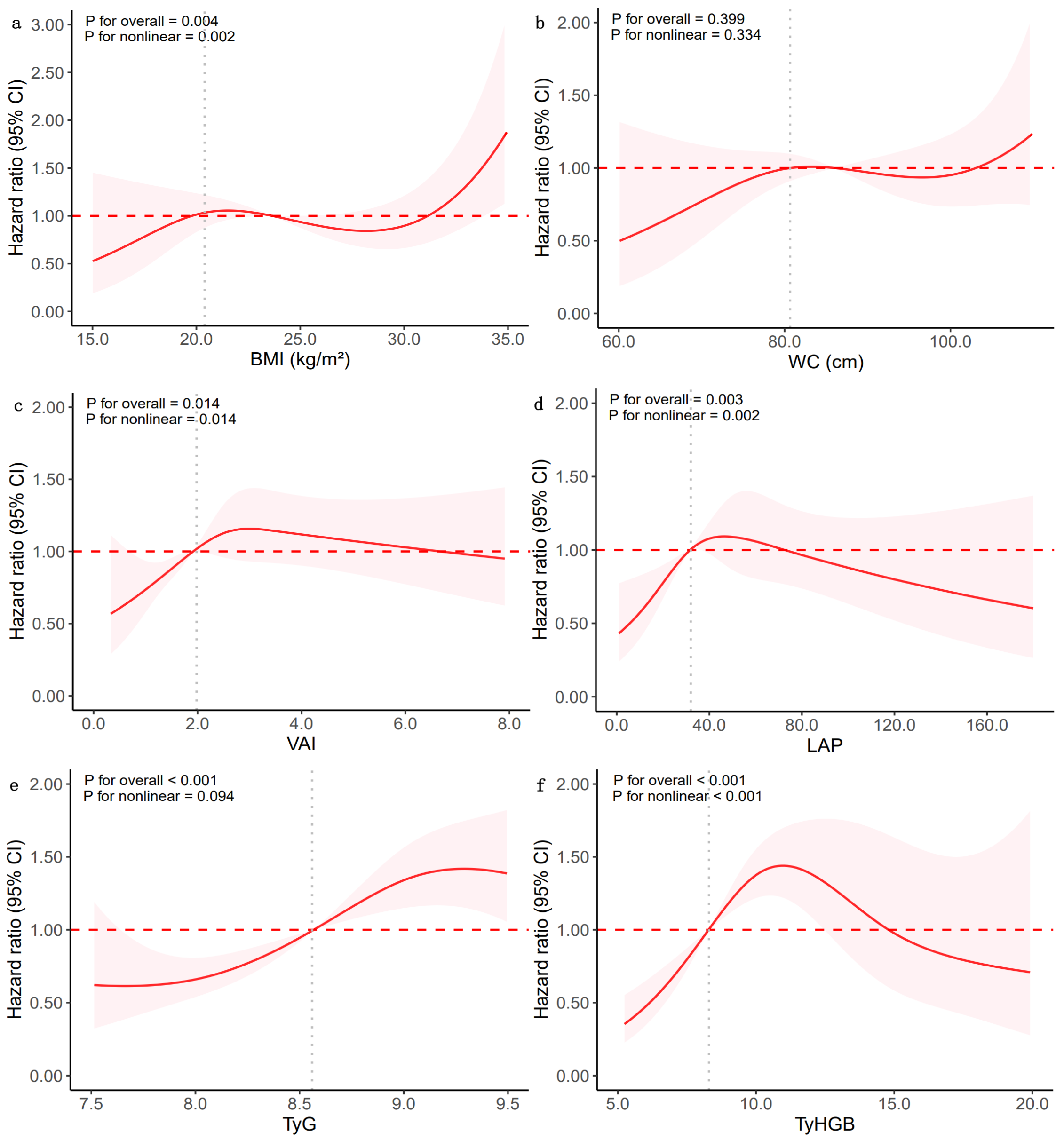

Supplement: Supplementary Figure 1 — Kaplan - Meier incidence rate of T2D according to quartiles of novel obesity- and lipid-related indices among normal FBG participants. (a) BMI, (b) WC, (c) VAI, (d) LAP, (e) TyG, (f), TyHGB. [file DataSheet1.zip › Figure S5.tif]

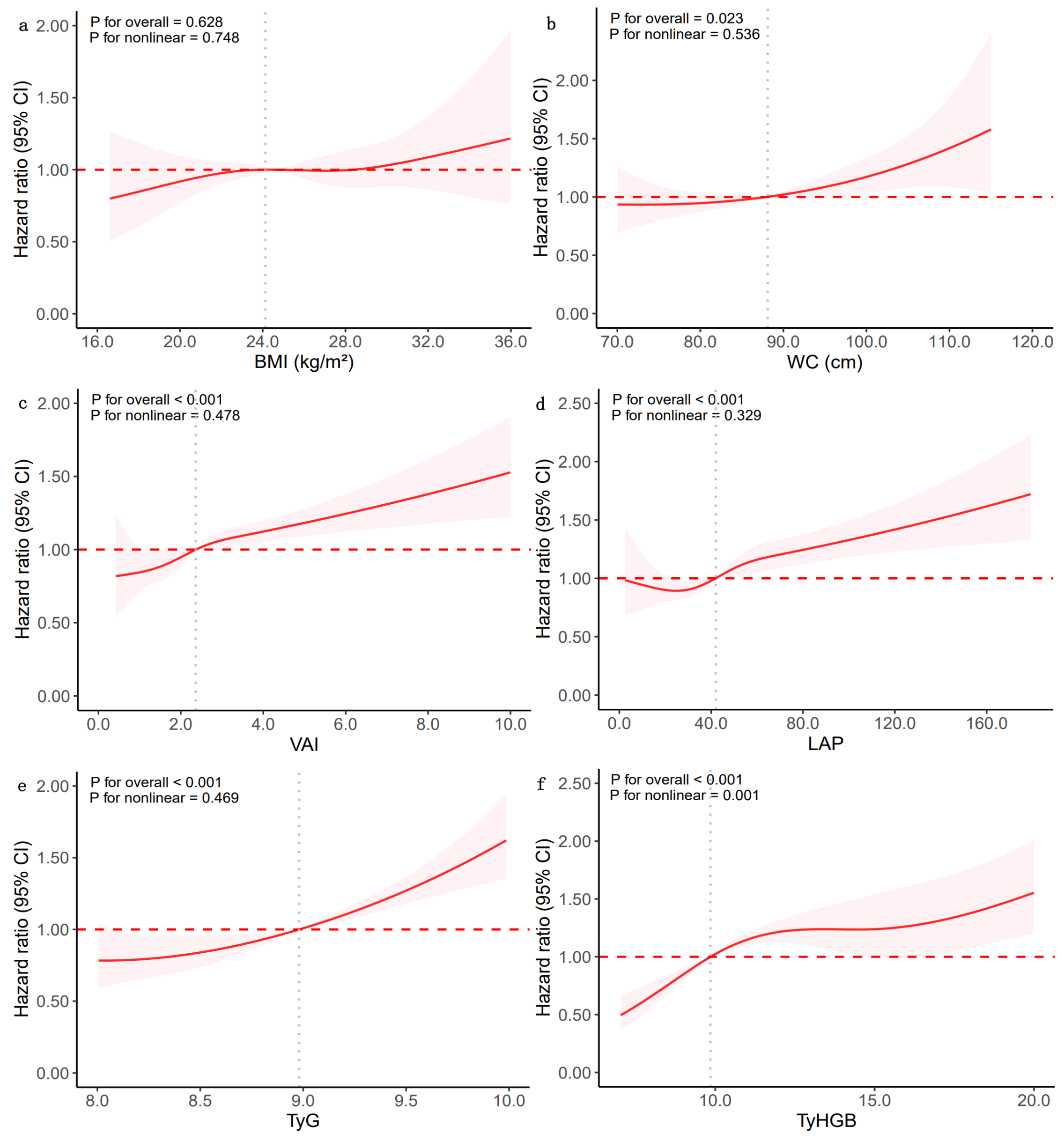

Supplement: Supplementary Figure 1 — Kaplan - Meier incidence rate of T2D according to quartiles of novel obesity- and lipid-related indices among normal FBG participants. (a) BMI, (b) WC, (c) VAI, (d) LAP, (e) TyG, (f), TyHGB. [file DataSheet1.zip › Figure S6.tif]

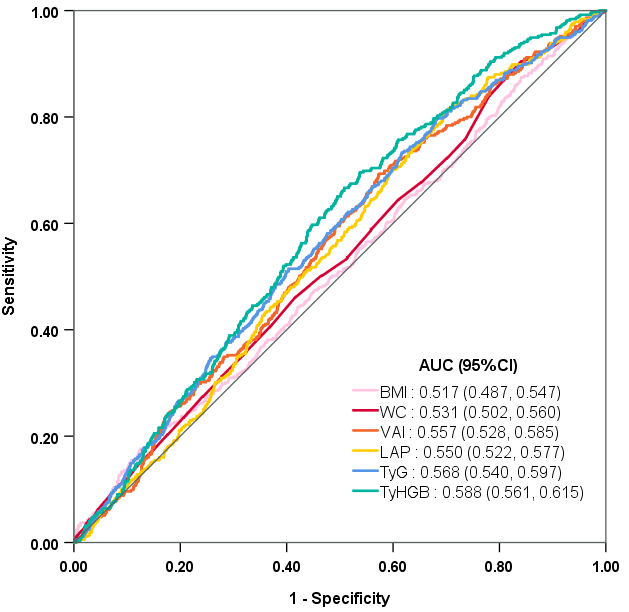

Supplement: Supplementary Figure 1 — Kaplan - Meier incidence rate of T2D according to quartiles of novel obesity- and lipid-related indices among normal FBG participants. (a) BMI, (b) WC, (c) VAI, (d) LAP, (e) TyG, (f), TyHGB. [file DataSheet1.zip › Figure S7.tif]

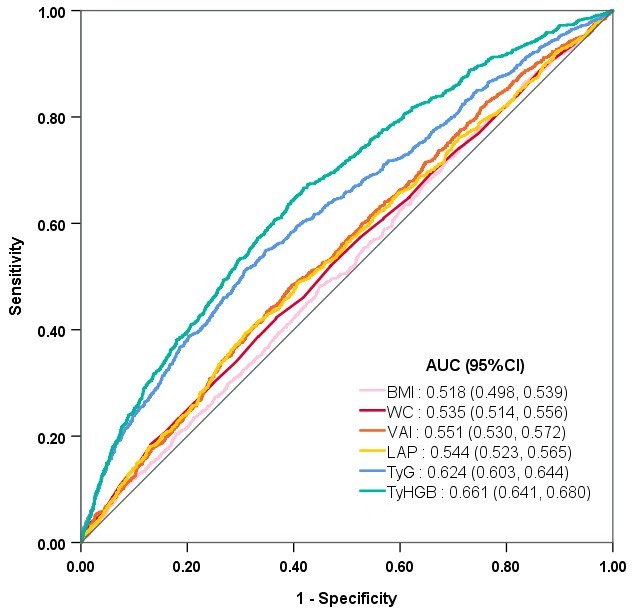

Supplement: Supplementary Figure 1 — Kaplan - Meier incidence rate of T2D according to quartiles of novel obesity- and lipid-related indices among normal FBG participants. (a) BMI, (b) WC, (c) VAI, (d) LAP, (e) TyG, (f), TyHGB. [file DataSheet1.zip › Figure S8.tif]
